# Factors Associated With Weight Change in Online Weight Management Communities: A Case Study in the LoseIt Reddit Community

**DOI:** 10.2196/jmir.5816

**Published:** 2017-01-16

**Authors:** Gisele Lobo Pappa, Tiago Oliveira Cunha, Paulo Viana Bicalho, Antonio Ribeiro, Ana Paula Couto Silva, Wagner Meira Jr, Alline Maria Rezende Beleigoli

**Affiliations:** ^1^ Computer Science Department Universidade Federal de Minas Gerais Belo Horizonte Brazil; ^2^ Internal Medicine Department Faculdade de Medicina Universidade Federal de Minas Gerais Belo Horizonte Brazil; ^3^ Centro de Telessaúde do Hospital das Clínicas Universidade Federal de Minas Gerais Belo Horizonte Brazil

**Keywords:** obesity, online social media, weight loss, user behavior, topic modeling

## Abstract

**Background:**

Recent research has shown that of the 72% of American Internet users who have looked for health information online, 22% have searched for help to lose or control weight. This demand for information has given rise to many online weight management communities, where users support one another throughout their weight loss process. Whether and how user engagement in online communities relates to weight change is not totally understood.

**Objective:**

We investigated the activity behavior and analyze the semantic content of the messages of active users in LoseIt (r/loseit), a weight management community of the online social network Reddit. We then explored whether these features are associated with weight loss in this online social network.

**Methods:**

A data collection tool was used to collect English posts, comments, and other public metadata of active users (ie, users with at least one post or comment) on LoseIt from August 2010 to November 2014. Analyses of frequency and intensity of user interaction in the community were performed together with a semantic analysis of the messages, done by a latent Dirichlet allocation method. The association between weight loss and online user activity patterns, the semantics of the messages, and real-world variables was found by a linear regression model using 30-day weight change as the dependent variable.

**Results:**

We collected posts and comments of 107,886 unique users. Among these, 101,003 (93.62%) wrote at least one comment and 38,981 (36.13%) wrote at least one post. Median percentage of days online was 3.81 (IQR 9.51). The 10 most-discussed semantic topics on posts were related to healthy food, clothing, calorie counting, workouts, looks, habits, support, and unhealthy food. In the subset of 754 users who had gender, age, and 30-day weight change data available, women were predominant and 92.9% (701/754) lost weight. Female gender, body mass index (BMI) at baseline, high levels of online activity, the number of upvotes received per post, and topics discussed within the community were independently associated with weight change.

**Conclusions:**

Our findings suggest that among active users of a weight management community, self-declaration of higher BMI levels (which may represent greater dissatisfaction with excess weight), high online activity, and engagement in discussions that might provide social support are associated with greater weight loss. These findings have the potential to aid health professionals to assist patients in online interventions by focusing efforts on increasing engagement and/or starting discussions on topics of higher impact on weight change.

## Introduction

Obesity is a major public health problem that adversely impacts morbidity, mortality, and quality of life. According to the World Health Organization (WHO), the prevalence of obesity has nearly doubled over the last 30 years [1]. According to a survey conducted by the Pew Research Center in September 2013 [2], of the 72% of American Internet users that have looked for health information online, 22% have searched for help to lose or control weight. These numbers show that people are seeking health advice and support online, especially in topic-driven communities and forums, Q&A sites (eg, Quora), and social media, including Twitter, Facebook, and Reddit [3,4].

Along with the growth of online searches for health advice, there has been a shift of social interactions from the real to the virtual world with the popularization of online social networks [5]. Most of these networks consist of communities organized according to user interests, including obesity and weight management groups. In contrast with real-world social groups, online social networks have no time restrictions, may be accessed from anywhere at no cost, and provide a constant source of information, support, and advice [6].

Among the factors that play a major role on the success of weight loss in online social networks are individual features and social embeddedness. Social embeddedness encompasses the structure and intensity of individual connections—reflecting different forms of engagement and participation in the community [3,5]—and it has been investigated across different weight management online communities [6-8]. Although social embeddedness has shown to strongly correlate with weight loss, most studies have mainly looked at the structural connections of the networks (ie, who connects to whom) [9]. Topics discussed by engaged users may also be as important as their engagement in the community. The linguistic style and semantics associated with the topics discussed might also be important features because they provide insights on personality, attitudes, and behavior, which in turn correlate with health outcomes [10]. However, most analyses look at syntactic features of the text, including word counts or the presence of positive or negative constructs [11,12]. Concerning the semantics of the messages, only a few qualitative studies focusing on small groups of users were able to analyze it. This is mainly due to the difficulties of automatically extracting semantics from text.

Analyses focusing on the behavior of users and the syntactic meaning of their texts are becoming common practice, not only in online health communities, but also in other contexts where user behavior in online social media can provide relevant information, including customer behavior [13] and citizens’ engagement with politics [14]. For instance, many marketing-related studies have proposed different theoretical frameworks to help understand the behavior of customers in virtual brand communities [15,16] or to identify the reasons for customers engaging online [17]. These frameworks and associated reasoning, despite being similar to ours, are not directly applicable to the health context. This is because the reasons for patients to engage on health communities are different from those of customers in a brand community and, most importantly, the role of the community toward users in both contexts differs significantly. Health online social network users are motivated mainly by having the opportunity to talk to users going through the same experiences without feeling pressure from society because obesity is still stigmatized. Furthermore, weight-loss communities present a large opportunity for ordinary users and health agents to perform both direct and indirect mental health and social support interventions, improving users’ self-motivation to lose weight and increasing their self-esteem [18,19].

In this paper, we describe both the activity behavior and analyze the semantic content of the messages of the LoseIt (r/loseit) online Reddit weight-loss community and investigate whether these factors are associated with weight loss in this online social network using different inputs to a regression model.

## Methods

### Data Collection

Reddit is an online forum organized in subcommunities by areas of interest called subreddits. We used Python Reddit Application Programming Interface (API) Wrapper (PRAW) [20], a Python package that eases access to Reddit’s official API, to collect the dataset from a Reddit subreddit called “LoseIt” [21], which is a community in which people interact about weight-loss issues [22]. Reddit users may submit content, such as texts or direct links to other sites, both collectively referred to as “posts.” The community can then vote posted submissions up (upvotes) or down (downvotes) as a sign of “liking” or “disliking” it. Users can also reply to posts with comments. One interesting aspect of Reddit is the anonymity of user accounts. In this study, users were identified by their system log-in. Data on real-world characteristics (age, gender, anthropometric measures), online activity behavior, and linguistic style were collected in English-written posts, comments, and tags in LoseIt from August 2010 to November 2014 [23].

### Study Measures

**Figure 1:**
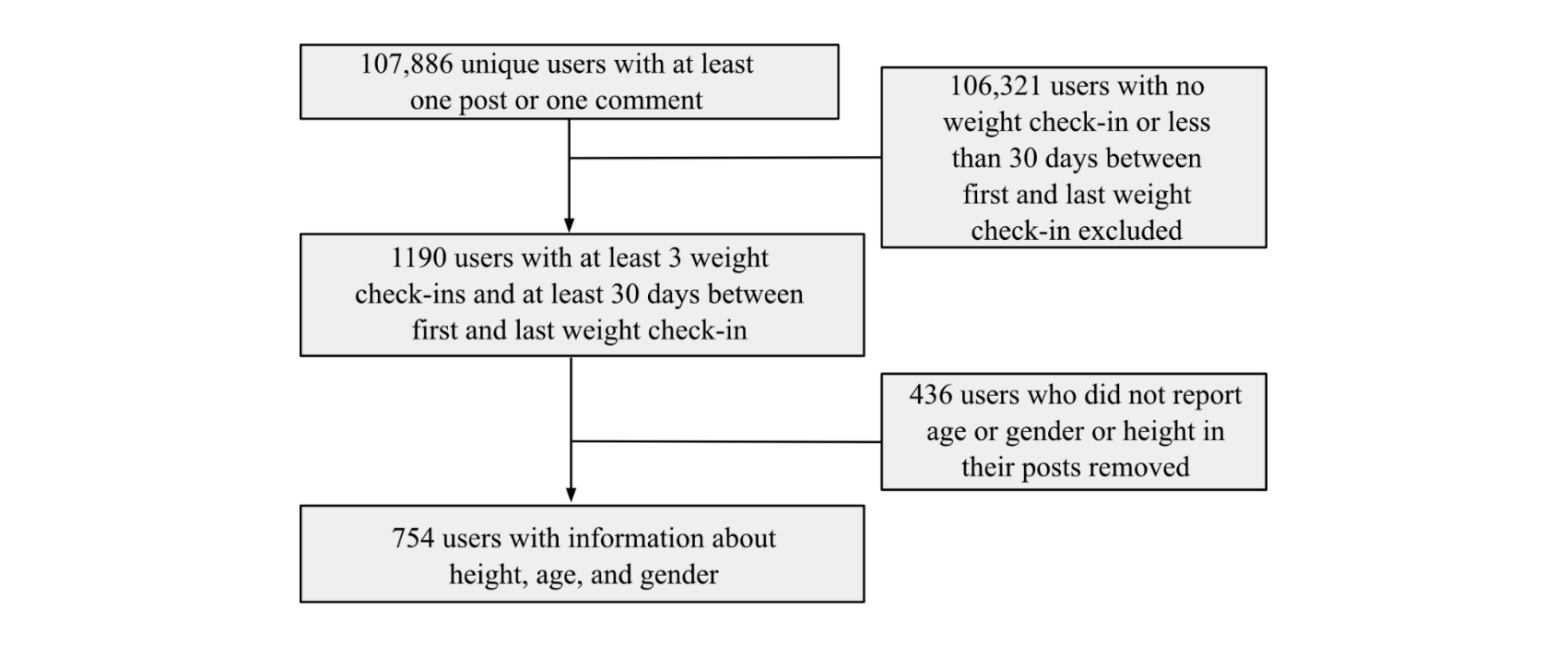
Flowchart of the user selection process.

Self-reports of weight, known as weight check-ins, included start weight, current weight, and goal weight. To deal with multiple reports of start weight (65/1190, 5.46%) per user, we selected the ones with the longer interval between start weight and current weight. To be considered for analysis, this interval had to be at least 30 days in an attempt to minimize the effect of short-term weight variations not related to fat loss on our outcome [24]. For a subset of users with this data available (n=1190), we collected data on age, gender, and height, and calculated body mass index (BMI) as BMI=weight (kg)/height (m^2^), obtaining a final sample of 754 users, as depicted in Figure 1. We categorized BMI according to the WHO criterion [25] of normal (BMI<25 kg/m^2^), overweight (25<BMI<30 kg/m^2^), class I obesity (30<BMI<35 kg/m^2^), class II obesity (35<BMI<40 kg/m^2^), and class III obesity (BMI≥40 kg/m^2^).

Measures of online activity were undertaken in relation to individual use (per user) and temporal activity. Data on the number of posts and comments (per time and per user), number of upvotes received per post and comment, number of comments received per post, and the number of weight check-ins were collected. We also computed the lifespan of a user, defined as the period of time between his/her first and last activity, and assessed the proportion of days online (days when a user posted or commented) and the number of active weeks. For assessing temporal patterns of activities, we derived a weekly temporal series for each user life span in LoseIt that estimated user participation over time (ie, we computed his/her number of activities in that week for each week in the lifespan of the user).

### Semantic Analysis of Text

After removal of special characters and stop words (the most common words in a language), we created a matrix of posts/comments per terms (words) using the term frequency [26]. This matrix was used as input to a semantic analysis of the messages through topics using latent Dirichlet allocation (LDA) with Gibbs sampling [27]. LDA assumes that there are hidden variables (topics) that explain the similarities between observable variables (posts or comments). The parameter for the number of topics was empirically defined as 50 and the method run for 2000 iterations. For each post/comment, LDA returns the probability of it being associated with a topic. Each topic, in turn, is represented as a set of words, in which each word is also associated with a probability of describing a topic. For each topic, we manually associated a label with the message given by its descriptive words and associated posts/comments.

### Statistical Analyses

Dataset characteristics were described by mean and standard deviation (SD), median and interquartile range (IQR), or frequencies.

We performed three linear regression models to investigate the association between weight change over at least 30 days and users’ real-world characteristics (model 1: age, gender, BMI), activity behaviors (model 2: number of activities, comments received, upvotes received, number of weight check-ins, lifespan, percentage of days active, number of days active, number of days offline, number of weeks active, and if the user has a verified email), and semantics extracted from text (model 3: the 26 most-discussed topics of posts and comments selected from a set of 100 topics using the Akaike information criterion) on weight change (over at least 30-day interval). For model 3, summed and normalized probabilities returned by the LDA for each user’s post or comment per topic were given as input to the regression model. Regression models were compared using the coefficient of determination (*R*^2^) and the adjusted *R*^2^, which also accounts for the number of variables included in the model.

To evaluate the generalization power of the models, we executed a 10-fold cross-validation [28]. This method divides the data into 10 nonoverlapping folds and uses nine parts to fit the model and one (also called test set) to measure its generalization performance. This procedure is repeated 10 times, each time with a different fold as the test. At the end of this process, the mean and standard deviation over the 10 folds are reported, together with the correlation between the real and predicted values in the user test set. All data were analyzed using R (MASS and caret packages).

## Results

### Data Sample Analysis

Over the 4-year follow-up, 107,886 of 252,279 (42.76%) Reddit users were active (ie, users with at least one post or comment). Among these, 101,003 (93.62%) wrote at least one comment and 38,981 (36.13%) wrote at least one post. For posts, 1621 of 107,886 users (1.50%) contributed more than five times, suggesting that this group starts several discussions around weight-loss problems. These users were the ones who remained active in the network for longer, with a median of 72 (IQR 78) active weeks versus a median of 1 (IQR 18) active weeks for the remaining users. We observed that more than 95.39% (64,281/67,387) of posts had at least one upvote. Considering the top 5.00% (3370/67,387) most upvoted posts, they all received more than 100 upvotes, suggesting that some posts do call the attention of the community and receive its support. Characteristics of the dataset are shown in Table 1.

**Table 1:** Overall characteristics of the LoseIt dataset.

Statistics	Median (IQR)	Mean (SD)
Posts per day	45.0 (30.0)	45.5 (22.7)
Posts per user	0 (1.0)	0.7 (1.8)
Comments per day	599.0 (333.0)	586.7 (264.3)
Comments per user	2 (4.0)	7.9 (34.4)
Upvotes per post	6.0 (16.0)	35.7 (126.7)
Upvotes per comments	2.0 (2.0)	3.1 (11.4)
Weight check-ins per user	2 (2.0)	2.7 (3.6)
Activities per user	2 (5.0)	8.5 (35.3)

For weight check-ins, 8834 of 107,886 (8.18%) users had at least one weight check-in. Among them, 6622 users declared a start weight, 6303 a current weight, and 3074 a goal weight. Among all users who declared a start weight, 65 (0.1%) declared it more than once, with 34 of 65 (52%) decreasing its value. The same happened with goal weights, which might indicate that 1.01% (31/3074) of the users reassessed their goal weight. In total, 19 of 31 users (62%) with more than one report reduced their initial goal weight, whereas 12 of 31 (38%) increased it.

### User Online Activity

**Figure 2:**
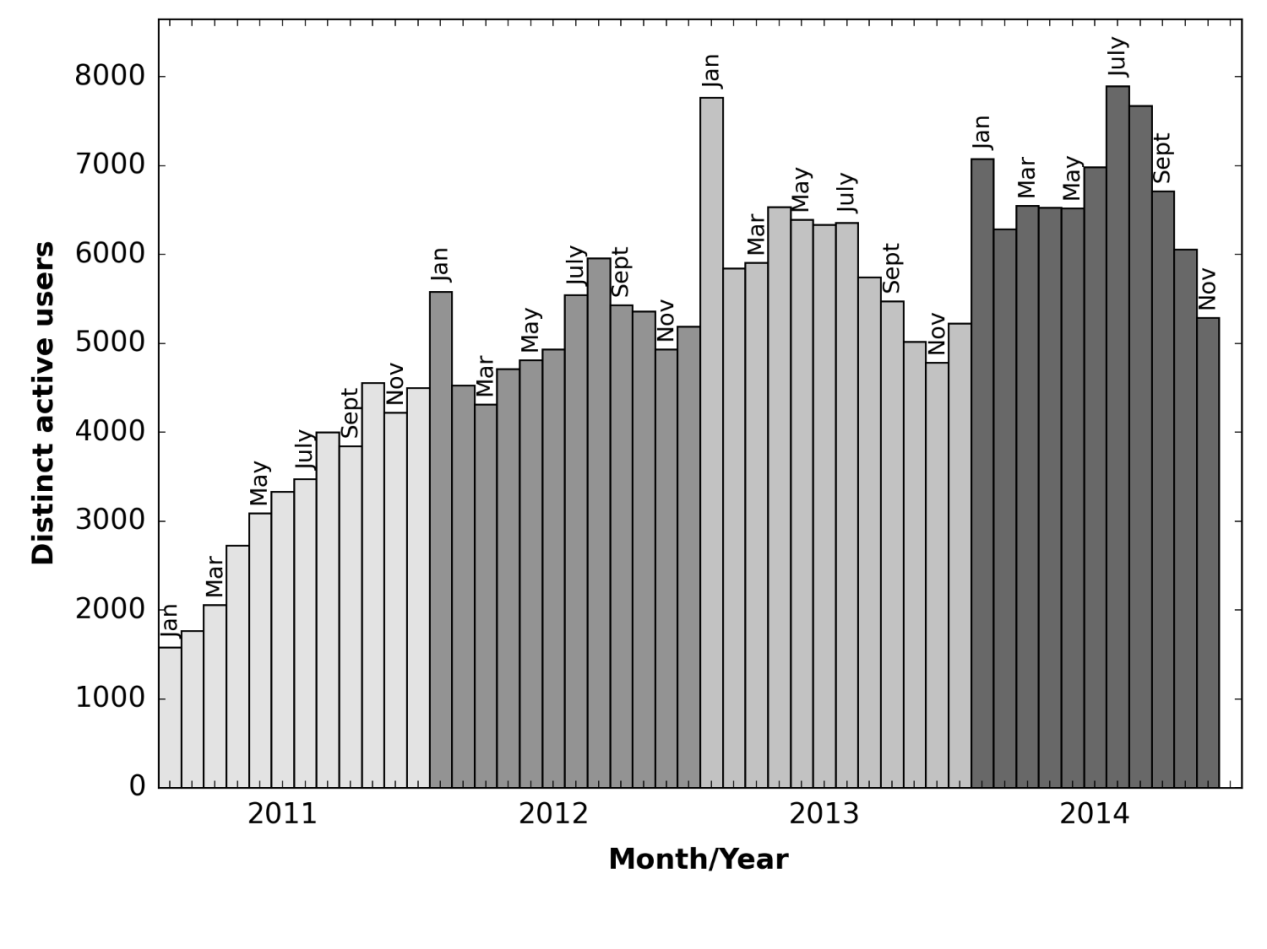
Number of distinct active users on LoseIt per month. Different colors correspond to different years.

Figure 2 shows the distribution of the number of distinct active users in the community over the follow-up period. Overall, the number of unique active users increased over time, with high values in January (motivated by New Year’s resolutions) and July to August (northern hemisphere summer).

Table 2 shows information about the user lifecycle. Considering the dates of the first and last activity, 50,705 of 107,886 (46.99%) users had a lifespan of one day, meaning they had a single activity in the network. Nevertheless, such short lifespans do not necessarily mean they left the community because they may have been simply acting as lurkers (ie, readers only). In contrast, 10,874 of 107,886 (10.08%) users had a lifespan longer than 1 year (449 days), and 543 of 107,886 (0.50%) reached 2 years (682 days). The longest life span was 1566 days (223 weeks). Disregarding the users who were active only once, for the 57,181 remaining users, the activity peak of 37,802 (66.10%) users was in week 1. Further, 45,403 of 57,181 (79.40%) users in the dataset reached their peak before week 10. In contrast, the peak for 3.54% (2024/57,181) of users was after week 40.

**Table 2:** Statistics about users’ lifecycles on LoseIt.

Lifecycle	Median (IQR)	Mean (SD)
Life span (days)	2 (132.0)	124.60 (238.64)
Percentage of days online	3.81 (9.54)	11.11 (17.97)
Weeks with activities	1 (2.0)	3.05 (5.09)
Peak of activity (week)	1 (0.0)	5.35 (15.88)

### Semantics Extracted From Text

Table 3 shows the 10 topics most frequently found in posts and messages, which are representations of the semantics of the posts. Note that by simply looking at the words describing topic 1, for instance, one might say they do not necessarily refer to healthy food, but the qualitative analysis together with the messages used allow us to state that. Also, notice that we may have more than one topic with the same label. This happens because the method used to extract the topics (LDA) does not guarantee they are unique. Examples of topics include information about workouts (topics 4 and 8) and the best ways to control calorie intake (topics 3 and 5). The topics in the comments are not that different from those in posts, although a strong presence of user support is frequently found in comments. The following comment illustrates the type of message we were dealing with:

Good work dude, I am in the same boat you are...I recently started strong lifts as well as the low-carb high-protein diet it’s awesome and I rarely feel hungry. Keep it up dude and when you feel comfortable post some pics. When I hit my first milestone of 50 lbs I will be posting some...hopefully soon down to go.

**Table 3:** Most frequently discussed topics in the LoseIt community for users’ (N=38,981) posts (n=67,387) and users’ (N=101,003) comments (n=771,146).

Given label		Top descriptive words	Posts, n (%)
**Posts**			
	1. Healthy food	Chicken, lunch, diner, breakfast, salad	3489 (5.18)
	2. Clothing	Size, fit, clothes, shirt, pant	2873 (4.26)
	3. Calories count	Calorie, day, counting, per, intake	2550 (3.78)
	4. Workout	Run, running, mile, minute, walk	2305 (3.42)
	5. Calories count	Using, use, mfp, track, myfitnesspal	1976 (2.93)
	6. Looks	Fat, body, muscle, stomach, skin	1952 (2.89)
	7. Habits	Water, drink, soda, drinking, cut	1933 (2.87)
	8. Workout	Cardio, workout, minute, training, lifting	1875 (2.78)
	9. Support	Loseit, thank, everyone, post, thanks	1831 (2.72)
	10. Unhealthy foods	Food, eat, pizza, one, ate	1803 (2.67)
**Comments**			
	1. Support	Great, look, awesome, job, amazing	44,829 (5.81)
	2. Food	Chicken, cheese, salad, vegies, cup	38,960 (5.05)
	3. Clothing	Size, fit, skin, clothes, loose	34,155 (4.43)
	4. Calories count	Calorie, day, counting, eat, deficit	21,458 (2.78)
	5. Calories count	Use, scale, track, using, mfp	21,081 (2.73)
	6. Weight control	Week, pound, month, lost, two	20,610 (2.67)
	7. Self-esteem	See, look, picture, progress, difference	20,588 (2.67)
	8. Workout	Run, running, minute, mile, walking	20,450 (2.65)
	9. Support	Feel, like, better, feeling, much	19,249 (2.49)
	10. Testimony	Self, not, problem, control, issue	19,232 (2.49)

### Understanding Weight Change

As showed in Figure 1, data on real-world characteristics were only available for 754 users; hence, the investigation of the factors associated with weight change is limited to this subsample. In this sample, 56.5% (426/754) of users were female and the mean age was 26 (SD 6) years. The mean difference between the start and goal weights was 35.6 (SD 24.8) kg; 3.7% (28/754) of users gained weight (mean 3.88%, SD 4.04), 3.5% (25/754) maintained weight, and 92.9% (701/754) lost weight. Overall, 514 of 754 users (68.2%) moved to a healthier category while participating in the LoseIt community as depicted in Table 4.

**Table 4:** User distribution according to initial and final obesity categories.

Initial category		Final category, n					
		Normal	Overweight	Obese I	Obese II	Obese III	Total
**Total**							
	Normal	25	2				27
	Overweight	70	58	1			129
	Obese I	37	105	46	1		189
	Obese II	10	52	76	32	1	171
	Obese III	4	36	61	63	74	238
**Female**							
	Normal	23	1				24
	Overweight	60	39	1			100
	Obese I	21	65	23	1		110
	Obese II	8	23	35	11	1	78
	Obese III	2	14	30	27	41	114
**Male**							
	Normal	2	1				3
	Overweight	10	19				29
	Obese I	16	40	23			79
	Obese II	2	29	41	21		93
	Obese III	2	22	31	36	33	124

Among weight losers, 350 lost up to 14.2% of their start weight and the top 10.0% (70/701) of weight losers lost between 29.4% and 57.7% of their start weight. Median weight loss was 41.6 (IQR 41.4) kg. In general, weight losers were active users; on average, they contributed mean 3.55 (SD 6.11) posts, made mean 59.21 (SD 164.01) comments, and received mean 50.26 (SD 106.05) comments.

Table 5 shows the regression coefficients found in the models for different variables along with their standard errors (SE) and *P* values. A positive coefficient indicates the variable is correlated to an increase in weight loss, whereas a negative coefficient indicates the variable is related to a decrease in weight loss. The *P* value for each term tests the null hypothesis that the coefficient is equal to zero (no effect). A low *P* value (<.05) indicates that you can reject the null hypothesis (ie, the predictor is likely to be a meaningful addition to the model because changes in its value are related to changes in the response variable).

**Table 5:** Variable coefficients (and their standard errors) found by three linear models accounting for different factors associated with percentage weight loss.

Variable		Model 1		Model 2		Model 3	
		Coefficient (SE)	*P* value	Coefficient (SE)	*P* value	Coefficient (SE)	*P* value
**Real world**							
	BMI	0.517 (0.090)	<.001	0.415 (0.090)	<.001	0.371 (0.044)	<.001
	Gender	–1.357 (0.140)	<.001	–1.979 (1.570)	<.001	–1.471 (0.781)	<.001
	Age	0.235 (1.630)	.88	0.126 (0.140)	.36	0.118 (0.069)	.09
**Online behavior**							
	Activity			–0.008 (0.005)	.11	–0.006 (0.001)	.01
	Comments received			0.120 (0.007)	<.001	0.005 (0.003)	.12
	Upvotes received			0.002 (0.001)	.04	0.002 (0.000)	<.001
	Number of weights			0.270 (0.120)	.02	0.288 (0.058)	<.001
	Weeks active			0.008 (0.070)	.90	0.089 (0.033)	<.001
	Off days			–0.004 (0.002)	.04	–0.004 (0.001)	<.001
**Post topics**							
	Weight control apps					–57.26 (26.68)	.03
	Self-esteem (1)^a^					118.50 (24.49)	<.001
	Self-esteem (2)^a^					48.03 (27.47)	.08
	Workout					37.22 (20.05)	.06
	Friendship					83.72 (28.31)	.003
	Health information					44.84 (31.49)	.15
	Weight goals					77.90 (26.99)	.004
	Feelings					–125.00 (37.61)	<.001
	Asking for advice					68.97 (44.70)	.12
	Motivation					68.51 (52.50)	.009
	Weight check-ins					42.02 (26.25)	.10
	Family					–47.16 (30.94)	.12
**Comment topics**							
	Body transformations					112.60 (59.71)	.06
	Asking for help					235.70 (96.420)	.01
	Body measures					–290.90 (108.20)	.007
	Changing lifestyle					–219.20 (141.40)	.12
	Counting calories					187.40 (71.55)	<.001
	Asking for support					–134.20 (63.51)	.03
	Friendship					223.40 (105.20)	.03
	Workout					295.30 (72.43)	<.001
	Motivation (1)^a^					304.80 (149.60)	.04
	Weight goals					243.00 (66.50)	<.001
	Motivation (2)^a^					251.80 (100.50)	.01
	Healthy foods					173.70 (91.41)	.05
	Gratitude					160.20 (72.06)	.02
	Starting again					–231.40 (106.10)	.02
	Lifestyle change					221.00 (99.84)	.03

^a^ Topics generated by latent Dirichlet allocation may overlap. The posts and comments for both self-esteem and motivation appear in two different topics, here indicated by (1) and (2) after their names.

Regarding the association between weight change and real-world characteristics, online activity behavior, and the semantics from text, we found that a higher initial BMI, higher number of weight check-ins and upvotes, the amount of time spent in the community, and the topics of comments and posts (self-esteem, friendship, motivation, asking for help, counting calories, workout, gratitude) were directly and independently associated with weight loss. Conversely, some topics (weight control apps, feelings, body measures, asking for support) were inversely associated with weight loss.

The addition to variables related to online activity behavior and topics to the model led to an improvement on its ability to explain weight loss (from *R*^2^=.18 in model 1 to *R*^2^=.26 in model 2 and from *R*^2^=.26 in model 2 to *R*^2^=.39 in model 3, respectively). The adjusted *R*^2^ did not change for models 1 and 2, and decreased from .39 to .36 in model 3. As assessed by the cross-validation procedure, our final model showed generalization power and robustness with an *R*^2^ mean of 0.39 (SD 0.01) and a weight loss correlation of 0.83 (SD 0.01).

## Discussion

Knowing about characteristics, experiences, and interests of users might be of great value for improving weight-loss strategies in online communities. Our analyses have shown that the LoseIt users’ activities are usually concentrated in their first 10 weeks in the community, when they are probably most motivated with the weight-loss process. Most of the active users reported that they lost weight, with approximately 70% moving to a lower obesity category. In addition, we found that users who reported weight change within a 30-day minimum interval were predominantly women, who declared themselves as overweight or obese. Female gender, BMI at baseline, high levels of online activity and of support (as measured by upvotes), and specific topics discussed within the community independently determined weight loss.

The predominance of women in comparison to men in our study may reflect both the higher prevalence of overweight and obesity among women in the general population [29] and a greater awareness of excessive weight among women in comparison to men [30]. Moreover, it may reflect the fact that women are more likely to pursue weight control [30,31] and to use the Internet to search for health information than men [32]. However, male sex was associated with greater weight loss. In real-world interventions, gender differences in success rates following short-term weight-loss programs are controversial [33], despite males’ higher metabolic and lipolytic rates [34]. In practice, these differences in gender composition of online communities might suggest that several dimensions of weight history that are known to be different between men and women should be considered for customizing apps for weight loss. For instance, the online community can be programmed to approach emotional eating (in response to mood) and social eating (eating in social situations) differently according to the gender of the user because emotional eating has been reported to be more common in women and social eating more common in men [33].

In this study, BMI was positively associated with weight loss. Because weight and BMI were self-reported, we might imply that the higher the baseline BMI, the greater the perception of excessive weight and/or dissatisfaction with weight status. Weight perception accuracy and dissatisfaction with weight status have been consistently associated with trying to lose weight and better weight control among obese and overweight adults in population-based studies [35]. However, due to the cross-sectional nature of our study, it is not possible to establish causality between self-declaration of high BMI and successful weight loss.

The number of weight reports, which may be a proxy of regular weight automonitoring, was directly associated with weight loss. Regular self-weighing has been reported as a useful tool for weight loss because it might provide feedback on energy balance status and consequent improvement in self-regulation [36]. It is not possible to establish a causal link between more frequent reporting and losing weight, but because individuals with greater weight loss might be more motivated to report their weight, it has been reported that self-reports of weight are more accurate among those who lose weight than among those who do not [37].

Various measures of online activity were independent predictors of weight loss. Higher participation levels in the online social network might unveil higher levels of self-motivation, which has been associated with better weight-loss outcomes following real-world interventions as well [38]. Therefore, developing strategies that improve self-motivation and maintain users active in the online community should be a cornerstone of Web-based weight-loss apps. These strategies might involve a more personalized approach with a deeper understanding of the uniqueness of the situation and needs of each user. In contrast with real-world situations, it has been reported that, among patients who seek health care for weight loss, the perception of the individual’s needs by health professionals was associated with higher rates of weight loss and adoption of healthy lifestyle habits [39]. Understanding that users have different demands in regard to the various types of social support [40], and developing computational algorithms to recognize these differences, might be key points to offer personalized assistance and enhance users’ activity in online social network.

Patients report that nonjudgmental and empathic interactions with health professionals are key points to achieve success in weight-loss programs in the real world [41]. Upvotes in online communities might lead to a sense of social belonging and improve perceived empathy of the user within the community [40]. This probably explains the positive association we found between the number of upvotes and weight loss. A satisfactory interaction between users can be accomplished in an online social network by moderation of posts and comments that might be stigmatizing or demotivating and implementation of rating systems that allow users to rate the usefulness of posts and comments.

The finding that adding topics discussed in posts and comments to the model that explains weight change led to a great improvement in the coefficient of determination of the model suggests that an online community should provide different types of social support to users to be effective because most topics reflect some kind of support. Social support in health communities has been shown to be associated with maintenance of health behavior change [42] and better weight-loss outcomes in real-world interventions [42,43]. Most of the topics that were significant determinants of weight loss can be associated with three of the four types of social support. Emotional (ie, provision of empathy) and/or appraisal (ie, provision of information that is useful for self-evaluation purposes) support might have been provided to users in posts and comments that discussed self-esteem, motivation, and friendship, for example. Informational (ie, provision of advice, suggestions, and information that may be used to solve problems) and appraisal support might have been provided by discussions regarding counting calories, body transformations, workout, healthy food, lifestyle changes, asking for help, and gratitude. Implementing computational strategies to recognize the topics discussed by users and making active efforts to maximize the engagement of users in forums that address these topics might be effective tools in online weight-loss programs.

A few topics we expected to show positive association with weight loss, such as weight control apps, body measures, feelings, and asking for support, showed a negative association to it. Regarding weight control apps, we can infer that users who talked about them in LoseIt might not have followed the interventions of the app appropriately because they may not have decided yet which weight control app to use. Concerning body measures, users talking about it might have been frustrated by their results or might have set unrealistic weight goals. Both conditions might be associated with less successful weight loss. Users who talked about feelings or asked for support probably did not feel appropriately supported, which might be related to unsuccessful weight loss.

We acknowledge some limitations of our study. The exclusion of less-engaged LoseIt users might have introduced a selection bias, which limits the generalization of the findings to active users of a closed online social network. Although it is not possible to exclude the possibility that unique users have multiple system log-ins, this issue probably did not influence our weight loss analyses due to the large sample size. Additionally, as in other studies with online communities, we relied on self-reports of weight, which may have reduced accuracy. The use of regular expressions for obtaining weight check-ins and real-world variables limited our data extraction and precluded us from having these data from the complete dataset. Furthermore, it was not possible to evaluate the influence of other factors that might impact LoseIt users’ weight change, such as dieting and exercise planning. Due to the cross-sectional nature of the study, it is not possible to infer causality between the investigated factors and weight loss.

As major strengths, we must highlight the use of a quantitative methodology, which automatically looks at our measures of interest and allows for an analysis of a big sample, the long-term follow-up of the social network, and the investigation of the influence of real-world, online behaviors and semantics of online discussions on weight change reported by users of the online social network. We focus on a content-centered community instead of a user-centered one (ie, users in Reddit do not have connections to people but to posts), where the role of semantics may be even more accentuated. The semantics, analyzed with LDA, is a good way to summarize the topics being discussed as well as represent the interests of individual users.

In conclusion, our findings show that users who are more active and engage in discussions that provide emotional, informational, and appraisal social support are the ones with more successful weight loss in the online social network LoseIt. With increasing access worldwide to computers, apps, and the Internet, as well as the substantial amount of health care resources demanded by the high prevalence of obesity—particularly at young ages—online communities might be important public health strategies for obesity treatment and prevention. To potentialize the benefit of these communities, specific features that increase online activity should be investigated and incorporated to the online social network. Furthermore, activities that stimulate social support among users might be key points in the planning of online weight-loss interventions.

## Abbreviations

API: application programming interface
BMI: body mass index
LDA: latent Dirichlet allocation
WHO: World Health Organization
